# A Novel Scale to Assess Humidification during Noninvasive Ventilation: A Prospective Observational Study

**DOI:** 10.1155/2023/9958707

**Published:** 2023-12-28

**Authors:** Longfang Pan, Yueling Hong, Xiaoqing Zhong, Jiao He, Zuli Zhang, Qianru Zhao, Linfu Bai, Mengyi Ma, Jun Duan

**Affiliations:** Department of Respiratory and Critical Care Medicine, The First Affiliated Hospital of Chongqing Medical University, Chongqing 400016, China

## Abstract

**Objective:**

To develop a novel scale to assess humidification during noninvasive ventilation (NIV).

**Methods:**

This study was performed in an ICU of a teaching hospital. Three ICU practitioners with more than 10 years of clinical experience developed an oral humidification scale with a range of 1–4 points. Each studied the current literature on humidification and examined 50 images of mouths of NIV patients with different levels of humidification. Then, through discussion, a consensus scale was developed. Next, 10 practitioners and 33 NIV patients were recruited to validate the scale. Finally, the patients rated the dryness of their mouths using the 1–4 visual scale just after the practitioners' assessment. Talking and discussion were forbidden during the assessment, and the scorers were blinded to each other.

**Results:**

We performed 36 assessments in 33 NIV patients. Three patients were assessed twice each more than 2 days apart. The interitem correlation coefficients between the 10 practitioners ranged from 0.748 to 0.917. Fleiss's kappa statistic was 0.516, indicating moderate agreement among practitioners. Of the 33 patients, 5 (15%) were unable to make an assessment using the 1–4 visual scale. Among the remainder, 55.7% provided scores that matched those given by the practitioners; 13.7% of scores were 1 point higher than that rated by the practitioners, and 20.7% were 1 point lower. Only 10% were beyond a 1-point difference. The kappa coefficient was 0.483 between patients and practitioners.

**Conclusions:**

The oral humidification scale showed moderate agreement between practitioners. It was also highly accurate in reflecting the level of humidification assessed by patients.

## 1. Introduction

Over the past two decades, the use of noninvasive ventilation (NIV) has significantly increased [1, 2]. This approach reduces the incidence of endotracheal intubation in patients with acute respiratory failure, facilitates ventilator weaning in patients on invasive mechanical ventilation, and improves survival in patients with chronic respiratory disorders [3–5]. Therefore, NIV has become a main oxygen strategy for patients with respiratory distress.

In normal respiration, the inhalation of room air is warmed and humidified by the upper airway to 37°C and 100% relative humidity [6]. However, in patients on NIV due to acute respiratory failure, the inspiratory flow, tidal volume, and minute ventilation increase dramatically and the demand for warming and humidification increases. In addition, medical air is generally much drier than room air, which further increases the need for humidification [7]. Inefficient humidification impairs mucociliary activity, which increases the risk of sputum retention, atelectasis, and pulmonary infection [6]. These factors play an important role in NIV failure [8].

Current guidelines recommend using active humidification in patients on NIV, as it improves compliance and comfort [9]. Heated humidification is frequently added to NIV patients [10]. However, inefficient humidification is not rare in NIV, even when the heated humidification is used [11]. Thus, frequent assessment of humidification during NIV is crucial to preserve airway moisture. However, there is currently no robust method for accurately assessing humidification during NIV. Therefore, we aimed to develop a novel scale to assess humidification during NIV for clinical reference.

## 2. Methods

**Figure 1:**
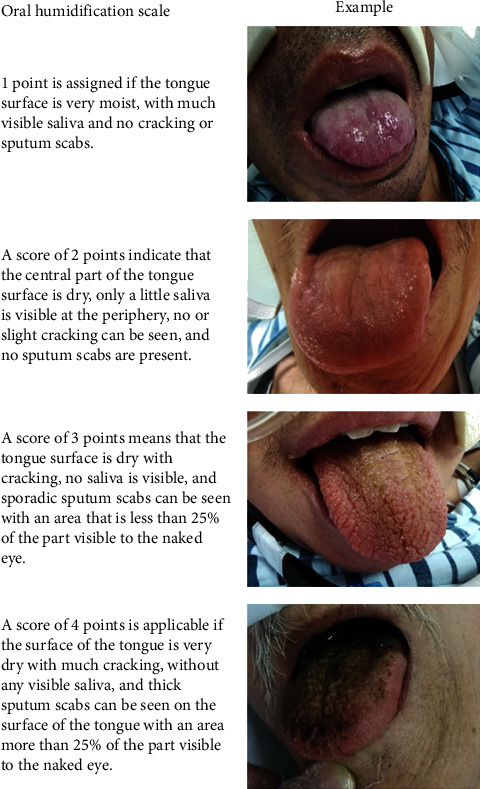
Description of the oral humidification scale.

This was an observational study performed in a respiratory ICU of a teaching hospital. Patients who used NIV with a face mask were enrolled. NIV was managed by the attending physicians, respiratory therapists, and the nurse in charge. All of the patients received a single-limb circuit and heated humidifier. The study protocol was approved by the applicable ethics committee (16/06/2021, No. 283). As this study was of an observational nature, the need for informed consent was waived. However, consent was obtained before taking pictures of the patients' tongues. The pictures selected for inclusion in Figure 1 were also approved by the patients themselves.

### 2.1. Development of the Oral Humidification Scale

Three ICU practitioners with more than 10 years of experience developed the humidification scale. First, they took photos of the tongues of patients who had been on NIV and selected 50 clear images showing different features. Then, they reviewed all of the pictures and developed the humidification scale in discussions together. The discussion mainly focused on the dryness of the lingual surface, the presence or absence of cracking, and the presence or absence of sputum scabs. The discussion is also referenced in the recent literature [12, 13].

After discussion, a 4-point scale was developed to assess humidification during NIV (Figure 1). The scoring was defined as follows: 1 point is assigned if the tongue surface is very moist, with much visible saliva and no cracking or sputum scabs; a score of 2 points indicates that the central part of the tongue surface is dry, only a little saliva is visible at the periphery, no or slight cracking can be seen, and no sputum scabs are present; a score of 3 points means that the tongue surface is dry with cracking, no saliva is visible, and sporadic sputum scabs can be seen with an area that is less than 25% of the part visible to the naked eye; and a score of 4 points is applicable if the surface of the tongue is very dry with much cracking, without any visible saliva, and thick sputum scabs can be seen on the surface of the tongue with an area more than 25% of the part visible to the naked eye.

### 2.2. Validation of the Oral Humidification Scale

To validate the humidification scale, 10 clinical practitioners who worked in ICUs were recruited and 33 patients who used NIV were also recruited by convenience sampling. First, the 10 clinical practitioners assessed the humidification for a given patient at the same time and assigned points, without discussion with their colleagues. Second, we asked the patient to report their sensation of dryness of the mouth on a scale of 1–4, where 1 is no dryness, 2 is mild dryness, 3 is moderate dryness, and 4 is severe dryness.

We collected demographic information of the 10 practitioners, including age, sex, education, technical title, occupation, and years of experience. We also collected the clinical features of the patients, including age, sex, diagnosis, underlying disease, and ventilator parameters. The ventilator parameters were the ventilation mode, inspiratory positive airway pressure, expiratory positive airway pressure, FiO_2_, air leak, tidal volume, minute ventilation, and measured respiratory rate.

### 2.3. Statistical Analysis

The data were analyzed using SPSS (version 25.0) and R (version 4.0.5). Continuous variables are reported as means and standard deviations or median values and interquartile ranges when appropriate. Grouped data are reported as proportions and percentages. A consistency test among the 10 practitioners was conducted using Fleiss's kappa statistic [14]. The strength of agreement was poor, slight, fair, moderate, substantial, and almost perfect for kappa coefficients of <0.00, 0.00–0.20, 0.21–0.40, 0.41–0.60, 0.61–0.80, and 0.81–1.00, respectively [15].

## 3. Results

In all, 33 patients who used NIV with a face mask were enrolled. Of these, three patients were assessed twice, more than 2 days apart. The demographic information of the enrolled patients is presented in Table 1. Their mean age was 73.7 years, 82% were male, and half were diagnosed with pneumonia. Before the assessment, the mean inspiratory positive airway pressure was 16.8 cmH_2_O, and the mean expiratory positive airway pressure was 7.6 cmH_2_O. The mean air leak during ventilation was 54.7 L/min, mean tidal volume was 542 mL, mean minute ventilation was 13.2 L/min, and mean measured respiratory rate was 25 breaths/min.

The demographic statistics of the 10 practitioners are presented in Table 2. Five were respiratory therapists, and five were ICU nurses. The mean age was 32.1 years, 40% of them were male, 50% of them had a bachelor's degree, and 50% of them had a primary technical title. The mean work experience was 11 years, and the mean work experience in ICU was 8.3 years.

Interitem correlation between different practitioners is summarized in Table 3. The correlation coefficient was between 0.748 and 0.917. Fleiss's kappa statistic was 0.516, indicating moderate agreement between practitioners.

**Figure 2:**
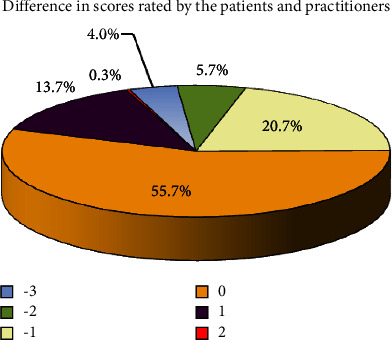
Percentage difference in scores rated by patients and practitioners.

Among the 33 patients, 5 (15%) were unable to provide assessments on a 1–4 visual scale of humidification. Among the remainder, 55.7% of scores rated by the patients were the same as the practitioners' ratings using the oral humidification scale; of the remainder, 13.7% were 1 point higher than that rated by the practitioners, and 20.7% were 1 point lower (Figure 2). Only 10% had more than a 1-point difference. The kappa coefficient between patients and practitioners was 0.483.

## 4. Discussion

To the best of our knowledge, this was the first study to develop a scale to assess humidification during NIV use. The scale showed moderate agreement among the practitioners. It also showed high accuracy in reflecting the level of humidification assessed by the patients.

Adequate humidification is important for patients during NIV intervention. It can reduce NIV-induced airway dryness, promote the transportation of secretions from the lungs, increase the tolerance of NIV, and improve patient comfort [16, 17]. However, inadequate humidification is not uncommon, even when heated humidification is used. During NIV intervention, oral breathing is less efficient than nasal breathing in humidification, increased air leak is associated with increased dryness of the mouth, and high inspiratory pressure decreases relative humidity [18, 19]. This significantly increases the probability of inadequate humidification. In routine clinical use, frequent checking of the need for humidification and appropriate adjustment of the heated humidifier is required. However, there are few simple methods to guide the delivery of humidification at bedside. This study provides a simple and reproducible method to assess the level of humidification during NIV intervention. It can serve as a convenient method to manage humidification in clinical practice.

It is difficult to measure the level of humidification. The use of an oral hygrometer is a quantitative method for such measurement [18]. It measures the percent weight of water in the oral mucosal epithelium based on the capacitance of the dielectric constant. Water content and the dielectric constant are positively correlated, and because the dielectric constant of water is markedly higher than that of other substances, the percentage of water in a substance can be determined by measuring its dielectric constant. However, this technique requires special equipment, which significantly limits clinical use. This study developed a semiquantitative scale for assessing the level of humidification based on the features of the tongue. Because the oral humidification scale is much easier to use than an oral hygrometer, it is a good choice for assessing humidification during NIV intervention.

Another method to assess the level of humidification is the use of a visual scale by the patients themselves [20, 21]. However, delirium is common in NIV intervention, with an incidence of 18% [22]. The visual scale cannot be used in such patients. In addition, it is time-consuming to teach a critically ill patient how to rate using a visual scale, particularly among aging patients. The oral humidification scale rated by the practitioners highly overlapped with the assessment ratings given by the patients themselves using a visual scale. This indicates that the oral humidification scale has sufficiently high accuracy to reflect the level of humidification. For patients who are unable to use a visual scale, the oral humidification scale is an alternative method to assess the level of humidification when NIV is used.

This study had several limitations. First, the dryness of the tongue can be influenced by drinking water. Assessment of dryness using this scale cannot be performed if the patient has had a drink of water before the assessment. Second, this was a single-center study. Other centers are encouraged to validate this scale. Third, the sample size was small. Further studies should seek to avoid this shortcoming.

## 5. Conclusions

Our proposed oral humidification scale showed moderate agreement between different practitioners. It also had high accuracy in reflecting the level of humidification assessed by the patients. As it was convenient and reproducible, this scale can serve as an alternative method for assessing humidification during NIV intervention.

## Figures and Tables

**Table 1:** Demographics of the enrolled patients.

Variables	Total cohort *N* = 33
Age, years	73.7 ± 12.1
Male (%)	27 (82%)
Diagnosis	
Pneumonia	16 (49%)
COPD	14 (42%)
Others	3 (9%)
Underlying disease	
Hypertension	18 (55%)
Coronary heart disease	7 (21%)
Pulmonary heart disease	6 (18%)
Diabetes	6 (18%)
Chronic heart failure	4 (12%)
Tumor	3 (9%)
Cerebral infarction	2 (6%)
Ventilator parameters^#^	
Ventilation mode	S/T
IPAP, cmH_2_O	16.8 ± 2.6
EPAP, cmH_2_O	7.6 ± 1.5
FiO_2_ (%)	39.4 ± 9.4
Air leak, L/min	54.7 ± 19.7
*V*_*T*_, mL/min	542 ± 159
MV, L/min	13.1 ± 4.2
Measured respiratory rate, breaths/min	25 ± 6

^#^We enrolled 33 patients and performed 36 assessments. COPD = chronic obstructive pulmonary disease, IPAP = inspiratory positive airway pressure, EPAP = expiratory positive airway pressure, *V*_*T*_ = tidal volume, MV = minute ventilation.

**Table 2:** Demographics of the 10 practitioners.

Variables	Raters (*N* = 10)
Age, years	32.1 ± 5.5
Male	4 (40%)
Working years	11.0 ± 4.9
Working years in ICU	8.3 ± 3.2
Education	
Bachelor's degree	5 (50%)
Master's degree	5 (50%)
Technical title	
Primary	5 (50%)
Intermediate	3 (30%)
Deputy senior rank	2 (20%)
Occupation	
Respiratory therapist	5 (50%)
ICU nurse	5 (50%)

**Table 3:** Interitem correlation matrix.

	Rater 1	Rater 2	Rater 3	Rater 4	Rater 5	Rater 6	Rater 7	Rater 8	Rater 9	Rater 10
Rater 1	1.000									
Rater 2	0.815	1.000								
Rater 3	0.849	0.813	1.000							
Rater 4	0.810	0.917	0.811	1.000						
Rater 5	0.815	0.807	0.839	0.861	1.000					
Rater 6	0.845	0.865	0.868	0.860	0.865	1.000				
Rater 7	0.778	0.751	0.837	0.791	0.833	0.769	1.000			
Rater 8	0.863	0.860	0.866	0.876	0.831	0.852	0.871	1.000		
Rater 9	0.820	0.815	0.820	0.835	0.733	0.784	0.789	0.846	1.000	
Rater 10	0.772	0.823	0.802	0.788	0.796	0.794	0.748	0.801	0.877	1.000

## Data Availability

The data used to support the findings of this study are available upon reasonable request to the corresponding author.
